# Systemic Administration of Rejuvenated Adipose-Derived Mesenchymal Stem Cells Improves Liver Metabolism in Equine Metabolic Syndrome (EMS)- New Approach in Veterinary Regenerative Medicine

**DOI:** 10.1007/s12015-019-09913-3

**Published:** 2019-10-16

**Authors:** Krzysztof Marycz, J. Szłapka-Kosarzewska, F. Geburek, K. Kornicka-Garbowska

**Affiliations:** 1 International Institute of Translational Medicine, Malin, Jesionowa 11 street, 55-114, Wisznia Mała, Poland; 2 Department of Experimental Biology, The Faculty of Biology and Animal Science, University of Environmental and Life Sciences Wroclaw, Wroclaw, Poland; 3 Faculty of Veterinary Medicine, Clinic for Horses - Department for Surgery, Justus-Liebig-University, Gießen, Germany

**Keywords:** Equine metabolic syndrome, Adipose derived stem cells, Liver, Cell therapy, Mesenchymal stem cells

## Abstract

Equine metabolic syndrome (EMS) is characterized by adiposity, insulin dysregulation and increased risk for laminitis. Increased levels of specific liver enzymes in the peripheral blood are typical findings in horses diagnosed with EMS. Current management of EMS is based on caloric restriction and increased physical activity. However, new potential treatment options are arising such as the transplantation of autologous adipose stem cells (ASC). However, cytophysiological properties of ASC derived from EMS horses are impaired which strongly limits their therapeutic potential. We hypothesized, that in vitro pharmacotherapy of those cells with 5-azacytidine (AZA) and resveratrol (RES) before their clinical application can reverse the aged phenotype of those cells and improve clinical outcome of autologous therapy. A 9 year old Dutch Warmblood Horse used for driving, was presented with severe obesity, insulin resistance. After EMS diagnosis, the animal received three intravenous injections of autologous, AZA/RES treated ASCs at weekly intervals. The therapeutic effect was assessed by the analysis of liver specific enzymes in the blood. ASC-transplantation reduced levels of glutamate dehydrogenase (GLDH), gamma-glutamyltransferase (GGT), lactate dehydrogenase (LDH) and aspartate transaminase (AST). This case report demonstrates the therapeutic potential of this intervention for EMS as well as apt utility of autologous, rejuvenated ASC injections.

## Introduction

Equine metabolic syndrome (EMS) has become more and more prevalent disorder affecting horses all over the world. EMS is characterized by the compilation of following factors: (i) regional adiposity in the neck, tail head and above the eye, (ii) insulin resistance and (iii) laminitis, both chronic and/or acute [1]. The disease is mainly caused by an inappropriate diet, and lack of exercise. Although there is growing interest in the study of EMS, there are difficulties regarding estimation of its prevalence. However, there is a solid, epidemiological data regarding occurrence of its components. For instance, the prevalence of obesity - one of the main risk factors for EMS- is estimated between 19 and 40% in domesticated equid populations [2, 3]. What is more, EMS-diagnosed animals are characterized by abnormal insulin response to oral glucose, hyperinsulinemia, hyperleptinemia, systemic and local inflammation, hypertriglyceridemia or mild triglyceridemia and dyslipidaemia. The authors´ previous study indicated on liver impairment in EMS horses at molecular level. Livers of those animals displayed signs of increased apoptosis and increased endoplasmic reticulum, oxidative stress, excessive accumulation of lipids and increased inflammation [4]. The authors´ unpublished data revealed that serum levels of aspartate aminotransferase (AST), alanine transaminase (ALT) and γ-glutamyl transferase (GGT) are increased in EMS horses. Still, to date no comprehensive data regarding liver condition in obese, insulin resistant horses with EMS is available. However, recent findings strongly supports the thesis, that liver may play a central and crucial role in the development of insulin resistance and EMS.

On the one hand dietary management is currently a first choice intervention for the treatment of EMS horses. Dietary protocol for EMS horses should include a low glycaemic index and high-fibre as well as low non-structural carbohydrate (NSC) contents. On the other hand, regular exercise is recommended as it not only helps to reduce body mass but also improves insulin sensitivity [5]. Metformin, a common drug for type two diabetes patients has been tested in EMS horses, however its oral availability was too low to exert expected therapeutic outcome [6]. Pioglitazone, which is applied in humans to treat obesity associated inflammation, did not lead to insulin sensitizing effects in horses [7]. Since no effective therapy for EMS exists, searching for a novel, valuable and effective strategy to increase insulin sensitivity in affected individuals is mandatory.

One of the key components of regenerative medicine are stem cells which help to repair injured tissues and enhance healing process. To date, most of ongoing clinical trials involve mesenchymal stem cells (MSCs) due to their unique properties: multilineage differentiation potential, immunomodulatory actions, ease of isolation and paracrine action through the synthesis of membrane derived microvesicles (MVs) rich in biologically active factors [8–11]. MSCs isolated from adipose tissue (ASCs) are widely applied in human and veterinary medicine as harvesting fat is easy and minimally invasive and results in high stem cells yield [12]. Due to their unique properties, ASCs hold great promise in the treatment of multiple disorders, including graft versus host disease, multiple sclerosis, autoimmune-induced diseases, acute liver disease, liver cirrhosis, non-alcoholic fatty liver disease, diabetes mellitus and EMS [13–17].

However, our previous data strongly indicated on the deterioration of cytophysiological and regenerative properties of ASCs isolated from EMS individuals (EMS-ASCs) [18]. In consequence, impairment of EMS-ASCs functionality questions their application in the treatment of EMS. Our results revealed that EMS-ASCs are characterized by decreased proliferation rate, increased apoptosis and senescence, oxidative stress and mitochondrial impairment as well as abnormal DNA methylation [19]. What is more, autophagy is triggered in those cells as a rescue type of mechanism allowing them to maintain multipotency. For that reason, novel approaches aiming to rejuvenate those cells in vitro before their clinical application are strongly desirable [20]. Especially in the context of recent findings which question immune privilege nature of MSCs suggesting that allogeneic cells may elicit immune response in recipient animals. In horses, formation of anti-allogeneic MSC antibodies, following an intradermal allogeneic MSC injection was observed [21, 22]. For that reason, we recently proposed in vitro pharmacotherapy of EMS-ASC in order to rejuvenate them before clinical application to make them an effective tool in autologous therapy. We cultured cells with the combination of 5-azacytidine (AZA) and resveratrol (RES) in order to reverse their aged phenotype. AZA was previously shown to decrease methylation in ASC derived from elderly donors, increase their proliferation rate [23] and enhance osteogenic differentiation [24]. On the other hand RES is a well-known antioxidant exerting immunomodulatory, anti-obesity, anti-inflammatory and anti-aging effects [25–28]. In our study we have shown, that AZA/RES combination can alleviate EMS-ASC deterioration and restore their physiological properties through modulation of mitochondrial dynamics and antioxidative action [29]. What is more, EMS-ASC treated with AZA/RES activated regulatory T cells (Tregs) [30].

This manuscript describe the case report of a horse diagnosed with EMS who was treated with AZA/RES treated autologous ASC. After three injections liver related parameters in the blood of animals of investigated in order to evaluate the potential of rejuvenated ASC in the modulation of liver metabolism.

## Materials and Methods

### Case Description

The study was performed after approval by the Local Ethic Committee in Wroclaw, Poland (84/2018). The patient was an 8 year old Dutch Warmblood gelding, who was diagnosed with EMS. The horse was used for combined driving purposes and competed on an international level since 3 years. A year before diagnosis, i.e. at the age of 7, the horse showed a gradually increase in weight until it became obese. The animal’s weight at 7 years old was 612 kg while body condition sore (BCS) [31] was estimated 7/8whith a cresty neck score (CNS) [32] of 4/5 At that time the horse showed lack of energy during work, especially extensive exercise and was weary. Then, for three days horse displayed symptoms of chronic laminitis in forelimbs. During that time, forelimbs were chilled and corn was excluded from the diet. For a week after first symptoms animal received analgesics and anti-inflammatory drugs. After occurrence of laminitis, oats was excluded from the diet and 3.5 kg of a commercially available low caloric feed was included in the dietary protocol (Brandon XL, Medvetico, Switzerland). Moreover, the horse received 100 g of commercially available additives (Glucogard,St. Hippolyt, Germany) daily. The diet was implemented 2 weeks before the first stem cells injection.

**Fig. 1:**
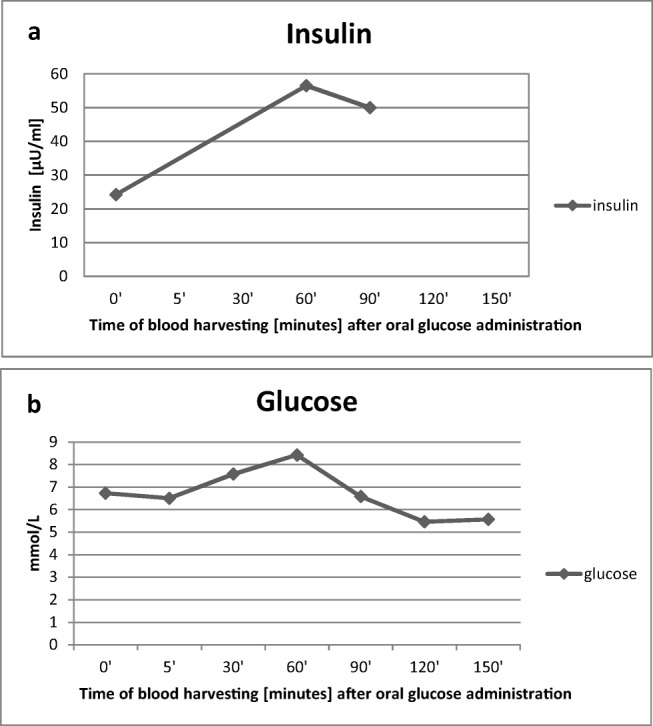
Results of the oral sugar test in an 8-year old Dutch warmblood horse with obesity. Plasma glucose (**a**) and plasma insulin (**a**) concentrations were measured over 150 min after oral administration of glucose

When the horse was 8 years old, an oral sugar test (OST) (Fig. 1) was performed according to a previously described protocol [33]. For that reason, the horse was completely fasted overnight, i.e. for 12 h while it had free access to water. In the following morning the OST was started with oral administration of 0.15 ml/1 kg BWT (bodyweight) of Light Corn Syrup (Karo) orally. Blood samples were collected from the external jugular vein using disposable needles. Blood was collected into plain tubes and tubes containing sodium fluoride and EDTA, respectively, immediately before (−5 min), and at 5, 30, 60, 90, 120 and 150 min after the oral administration of syrup. Sodium fluoride tubes were used to determine the blood glucose concentration. After collection tubes were gently inverted several times to mix blood and anticoagulant. Chilled samples were transported to the laboratory. The plain tubes without any additives (i.e. separator gels, clot activators, inhibitors, etc.) were used to collect blood for insulin level determination- for that blood was collected at 60 and 90 min after the syrup administration. The plain collection tube for serum was kept at room temperature for 15 min to avoid fibrin formation and ensure sufficient serum yield.

Samples were collected in serum or plasma (EDTA) tubes (by standard lab procedures. The following parameters were investigated during the study: blood cell count, glucose, insulin, glutamate dehydrogenase (GLDH), gamma-glutamyltransferase (GGT), lactate dehydrogenase (LDH) and aspartate transaminase (AST). Based on clinical findings and blood parameters obtained, it was decided to inject the horse with AZA/RES treated autologous stem cells in order to improve his liver function.

### Adipose Tissue Harvesting

As the OST was positive and the animal was diagnosed with EMS adipose tissue was harvest in order to isolate ASCs. The horse was sedated with 0.12 ml/100 kg BWT Cepesedan (detomidine hydrochloride, CP-Pharma Handelsgesellschaft, Scan Vet, Poland) and 0.25 ml/100 kg BWT of Torbugesic (Butorphanol tartrate 10 mg/ml, Zoetis, Belgia) intravenously. Then, the area around the base of the tail was washed, shaved and aseptically prepared. Then local subcutaneous analgesia was performed with 10 ml of a 2% lidocaine hydrochloride (lignocainum hydrochloricum WZF 2%, Polfa Warszawa S.A, Poland),). The skin was incised with a sterile scalpel and a cuboidal piece of adipose tissue (2 g) was removed from the subcutis using a tweezers and scissors. The skin incision was closed with simple interrupted sutures using the monofilament, synthetic, non- absorbable suture material polyamide, (Dafilon USP 1) while the adipose tissue was transferred to the laboratory in sterile Hank’s balanced salt solution (HBSS) supplemented with 1% PS on ice.

### Cell Isolation and Culture

Cells were isolated under aseptic conditions as described previously [34]. Briefly, the sample was extensively washed 3 times with HBSS, cut into small pieces and minced. The specimen was then digested in collagenase type I solution (1 mg/mL) for 40 min at 37 °C. Then the sample was centrifuged (1200×g, 10 min) and the supernatant was discarded. The remaining cell pellet was re-suspended in culture medium and transferred to a T25 culture flask. Prior to the experiments, cells were passaged 3 times. Culture medium consisted of DMEM with 1 g/L glucose (DMEM LG) supplemented with 10% fetal bovine serum (FBS) and 1% of penicillin-streptomycin (PS). After the third passage, the culture medium was exchanged for medium supplemented with 0.5 μM of AZA and 0.05 μM of RES. Cells were treated with AZA/RES for 24 h and then was detached from the dish, counted and prepared for injection. The molecular phenotype, ability to differentiate and multiple other characteristics of cells treated with AZA/RES were published by our group previously [19, 20].

Osteogenic differentiation, proliferative activity and population doubling time (PDT) of AZA/RES treated cells was performed as described previously [35]. JC-1 test and superoxide dismutase (SOD) assay were performed as described elsewhere [35].

### Injection of Cells

Prior to each injection, cells were suspended at the concentration 10 million/ml in physiological saline solution, transferred in a sterile syringe in the final volume of 3 ml and injected into a jugular vein. Stem cells were administered 3 times at weekly intervals.

## Results

### The Effects of AZA/RES on ASC

**Fig. 2:**
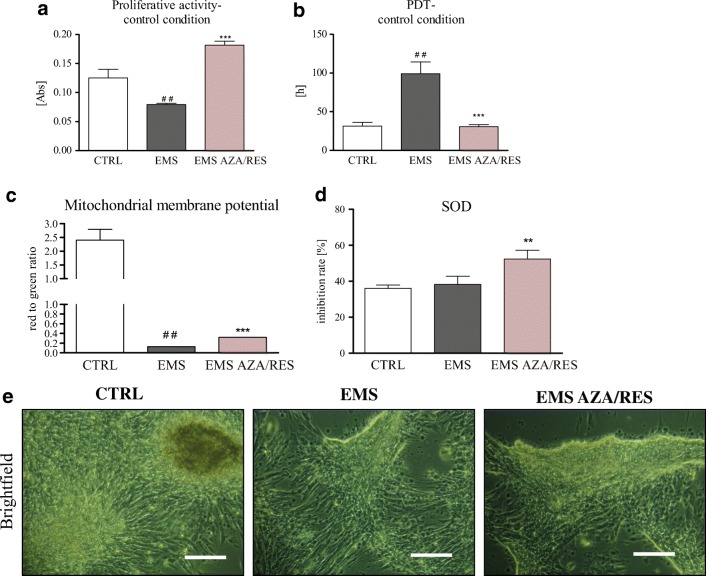
Alamar blue assay (**a**) was performed to determine the proliferation rate of cells (**a**). Untreated cells served as a control group (CTRL and EMS). PDT (**b**) was decreased while the mitochondrial membrane potential (**c**) increased in the experimental group. AZA/RES treatment increased SOD activity with (**d**) and enhanced osteogenic differentiation (**e**). Results are expressed as mean ± SD. Statistical significance is indicated as asterisk (*) when comparing the result to ASCEMS, and as hashtag (#) when comparing to ASC from healthy horse (CTRL). ##*P* < .01, **P < .01, ***P < .001. (Reproduced from Marycz et al. [35] under the Creative Commons Attribution License https://www.ncbi.nlm.nih.gov/pmc/articles/PMC6156237/)

Cells after AZA/RES treatment displayed an increased proliferation rate (Fig. 2a), PDT (Fig. 2b) increased mitochondrial membrane potential (Fig. 2c) and SOD activity (Fig. 2d). AZA/RES enhanced the formation of extracellular mineralized matrix during osteogenic differentiation in treated ASC EMS (Fig. 2e).

### Clinical Evaluation before and after Injections

**Fig. 3:**
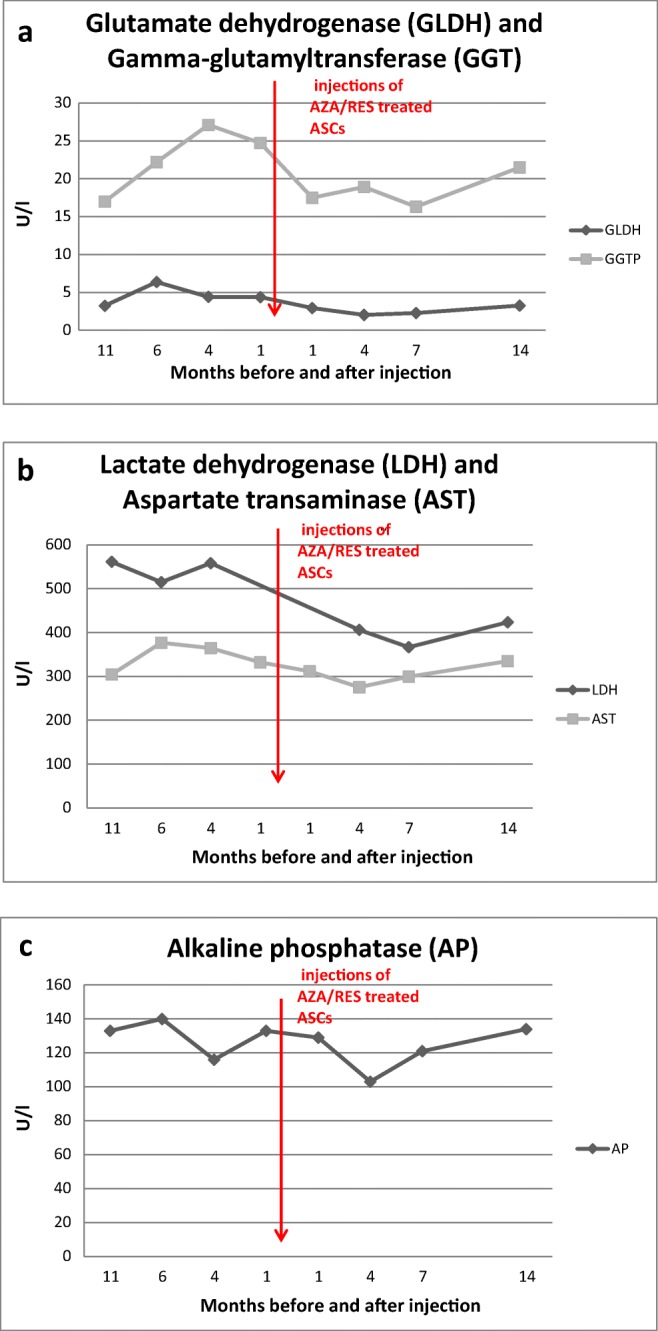
Evaluation of GLDG and GGT(**a**), LDH and AST (**b**) and AP (**c**) levels in the serum an 8-year-old Dutch warmblood horse before and after intravenous administration of AZA/RES treated ASCs

**Fig. 4:**
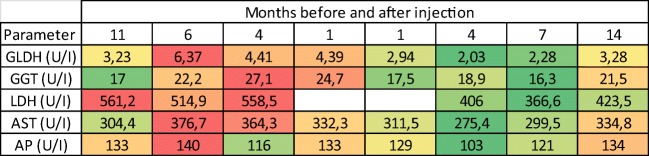
Results of the GLDH, GGT, LDH, AST and AP measurements in different time points shown as a heat map. Lower values highlighted in green highest in red

After the injections of AZA/RES treated ASCs, the horse behaved normally and did not exhibit any adverse reaction. After administration of ASCs, GLDH levels decreased, however, not as significantly as GGT did (Fig. 3a). A month after the last injection, the GGT level in the circulating blood decreased. GGT level was reduced until the 8th month after last injection. A similar trend was observed for LDH and AST levels which decreased in peripheral blood after ASC-injections. (Fig. 3b). LDH levels never reached a value above the one observed before ASC administration (Fig. 3b). AP levels decreased after ASC injections however, after 7th month they increased again. Results of GLDH, LDH, GGT, AST, AP presented in a table as a heat map (Fig. 4). Blood cells counts of the horse at multiple time points before and after ASC injections are shown on Table 1. No significant changes were noted after ASC therapy.

**Table 1:** Blood cells counts before and after injection

Parameter	Unit	Months before (b) and after (a) injections						Norm
		6 (b)	4 (b)	1 (a)	4 (a)	11 (a)	14 (a)	
WBC	G/l	6.81	7.93	5.74	7.15	7.81	8.07	5.0–10.0
NEU	G/l	4.4	4.47	3.58	4.19	4.55	5.4	3.0–7.0
LYM	G/l	2.02	2.95	1.79	2.44	2.47	2.15	1.5–4.0
MONO	G/l	0.296	0.382	0.199	0.419	0.576	0.326	0.04–0.40
EOS	G/l	0.065	0.1	0.128	0.06	0.18	0.139	0.04–0.35
BASO	G/l	0.029	0.029	0.042	0.052	0.024	0.054	0.001–0.15
RBC	T/l	10.3	10.2	5.78	8.19	7.07	8.46	6.0–12.0
HGB	g/dl	16.3	16.5	**88.6**	137	115	140	110.0–170.0
HCT	%	49.1	48.8	**0.29**	0.413	0.362	0.42	0.3–0.5
MCV	fl	47.8	47.7	50.2	50.4	51.2	49.6	37.0–55.0
MCH	pg	15.9	16.2	15.3	16.7	16.3	16.6	13.0–19.0
MCHC	g/l	333	33.9	306	331	318	334	310.0–360.0
PLT	G/l	115	111	79,7	111	52,4	74,2	90,0-500,0

WBC-white blood cells; NEU- neutrophils; LYM- lymphocytes; MONO- monocytes; EOS-eosinophils; BASO- basophils; RBC- red blood cells; HGB- haemoglobin; HCT- haematocrit; MCV- mean corpuscular volume; MCH- mean corpuscular haemoglobin; MCHC- mean cell haemoglobin concentration; PLT- platelets

ldh was not measured at that timepoints, so you can add *x* symbol inside those two cells

## Discussion

The efficiency of stem cells therapy in endocrinological disorders has long been discussed. Here, we have shown that AZA/RES treated, autologous ASCs can be applied effectively in horses, and have a great potential to improve liver metabolism deteriorated in the course of EMS. However, therapy was combined with proper dietary management and physical exercise as a standard treatment procedure during EMS which could also contributed to improvement of liver parameters. To date, not enough attention has been paid to the liver function of horses with EMS although it may play a key role in the development and progression of this disease.

Multiple studies have highlighted the efficiency of MSCs in the treatment of obesity, insulin resistance and hepatic and musculoskeletal disorders. Si et al. [36] revealed that MSC from bone marrow (BM-MSC) transplantation reduced hyperglycaemia in diabetic rats by activating the insulin receptor substrate (IRS)-1 signalling pathway. It also increased expression of glucose transporter 4 (GLUT-4) in insulin sensitive tissues ameliorating insulin resistance [36]. Not only ASCs but also their conditioned media have been shown to reverse insulin resistance via up-regulation of GLUT-4 expression and reductions in the expression of interleukin 6 (IL-6) and plasminogen activator inhibitor-1 (PAI-1) [37]. However, the mechanism by which MSCs ameliorate insulin resistance could not be understood completely. Utility of MSC in metabolic syndrome is supported by their anti-inflammatory properties, as local and/or systemic inflammation occurs during EMS. It was shown that MSCs from the human umbilical cord (UC-MSCs) alleviate insulin resistance in diabetic rats by reprogramming classically activated macrophages (M1, pro-inflammatory) into an alternatively activated M2 anti-inflammatory phenotype (M2, anti-inflammatory) [38]. Furthermore, MSCs promote the generation of regulatory T cells (Tregs), directly through the constitutive production of tumour growth factor beta one (TGF-β1) and indirectly by pathways involving the differentiation of monocytes towards CCL18 producing M2 macrophages [39]. Interestingly, in the authors´ previous study it was shown that AZA/RES treated EMS-ASCs induce the generation of Tregs more efficiently than untreated cells [30]. Thus, it is tempting to speculate, that after injections those cells exert similar effect in vivo. Increased numbers of circulating Tregs in EMS individuals may reduce the inflammatory state of the liver thereby contributing to the improvement of its metabolism. It is also postulated by some researchers that ASCs exert their immunomodulatory and anti-inflammatory effects mainly through their paracrine action, secreting MVs rich in cytokines and growth factors [40]. In the authors´ previous research it has been shown that AZA/RES treated EMS-ASCs secrete more MVs compared to untreated cells which justifies their application in EMS horses as a tool to diminish the inflammatory state in tissues and organs including the liver.

In previous studies it has been shown that MSC-injections enhance liver function, reduce hepatocyte apoptosis and promote their proliferation in mouse and rat models of acute liver failure [41, 42]. However, some studies indicated that systemically infused MSCs are blocked in the lung and short-lived and that no viable MSCs are found in other organs [43, 44].

In the case presented here, beneficial effect of ASC transplantation was maintained up to 7 months after injections. Interestingly, in humans with chronic liver diseases therapeutic effects of MSC administration could be detected up to 36th -38th week [45] and was indicated that re-administration prolongs the efficacy of MSC therapy. Following intravenous injection, MSCs initially travel to the lungs, followed by the liver, which supports their application in diseases related to a deterioration of the liver function and structure. In a pilot study, human patients with primary biliary cirrhosis were given MSCs three times at 4-week intervals and after 48 weeks a significant decrease in serum AP and GGT levels was observed. Interestingly, no changes in the levels of ALT nor AST were noted [46]. In dogs suffering from hepatocutaneous syndrome allogeneic ASCs were infused 46 times over a 30-month period (8 times directly into the liver and 38 times into peripheral veins). During this therapy all liver enzyme activities (ALT, AST, AP, GGT) gradually decreased [47]. Liu et al. revealed that injection of human UC-MSCs reduce hepatocyte apoptosis and enhance liver regeneration by paracrine pathways and reduce levels of inflammatory cytokines (TNF-α and IL-6) while increase serum hepatic growth factors (HGF) levels in mice [48].

Because of their unique properties, including immunomodulatory effects and multipotency, MSCs are increasingly becoming a useful medical tool for tissue regeneration [49, 50]. Multiple previous studies have shown that MSCs can be applied for the treatment of insulin resistance and liver failure. However, in most of the studies, the beneficial effect of MSCs was rather short, and not long-term. It is still unclear whether MSC-therapy can provide long-term benefits and if there is a way to prolong its therapeutic effects. In case of liver regeneration, MSCs may be particularly useful due to their anti-inflammatory properties and their ability to differentiate into hepatocytes [51]. To our knowledge this is the first report describing the effects of rejuvenated ASCs onto liver metabolism under EMS condition. We could show, that a triplicate ASC administration has the potential to decrease the activity of liver enzymes for up to 6 months. It should be mentioned that therapeutic effects are enhanced by conventional EMS management. A novel approach to treat EMS combines ASC-administration and supplementation of a horse diet containing bioactive molecules in the form of food additives [15]. As EMS is a complex, multifactorial disorder its management should address multiple levels. However, there are still, multiple concerns regarding MSC-therapy including the route of administration, dosage, time of application and safety of allogeneic transplantation. There is also a need to develop methods that improve the colonization rate, the survival rate of MSCs in vivo and enhance therapeutic effects.. In our opinion, treatment of EMS-ASC with AZA/RES represents one of those methods. Pharmacotherapy of MSC before their clinical application in order to strengthen therapeutic outcome may become a gold standard in personalized regenerative medicine.

## References

[1] Frank N (2009). Equine metabolic syndrome. Journal of Equine Veterinary Science.

[2] Wyse CA, McNie KA, Tannahill VJ, Tannahil VJ, Murray JK, Love S (2008). Prevalence of obesity in riding horses in Scotland. The Veterinary Record.

[3] Stephenson HM, Green MJ, Freeman SL (2011). Prevalence of obesity in a population of horses in the UK. The Veterinary Record.

[4] Marycz K, Grzesiak J, Wrzeszcz K, Golonka P (2012). Adipose stem cell combined with plasma-based implant bone tissue differentiation in vitro and in a horse with a phalanx digitalis distalis fracture: A case report. Veterinarni Medicina (Czech Republic).

[5] Powell DM, Reedy SE, Sessions DR, Fitzgerald BP (2002). Effect of short-term exercise training on insulin sensitivity in obese and lean mares. Equine Veterinary Journal.

[6] Hustace JL, Firshman AM, Mata JE (2009). Pharmacokinetics and bioavailability of metformin in horses. American Journal of Veterinary Research.

[7] Wearn JMG, Crisman MV, Davis JL, Geor RJ, Hodgson DR, Suagee JK (2011). Pharmacokinetics of pioglitazone after multiple oral dose administration in horses. Journal of Veterinary Pharmacology and Therapeutics.

[8] Marycz Krzysztof, Mierzejewska Katarzyna, Śmieszek Agnieszka, Suszynska Ewa, Malicka Iwona, Kucia Magda, Ratajczak Mariusz Z. (2016). Endurance Exercise Mobilizes Developmentally Early Stem Cells into Peripheral Blood and Increases Their Number in Bone Marrow: Implications for Tissue Regeneration. Stem Cells International.

[9] Oreffo ROC, Cooper C, Mason C, Clements M (2005). Mesenchymal stem cells. Stem Cell Reviews.

[10] Holan V, Hermankova B, Bohacova P, Kossl J, Chudickova M, Hajkova M, Javorkova E (2016). Distinct Immunoregulatory mechanisms in mesenchymal stem cells: Role of the cytokine environment. Stem Cell Reviews and Reports.

[11] Abumaree M, Al Jumah M, Pace RA, Kalionis B (2012). Immunosuppressive properties of mesenchymal stem cells. Stem Cell Reviews and Reports.

[12] Cislo-Pakuluk A, Marycz K (2017). A promising tool in retina regeneration: Current perspectives and challenges when using mesenchymal progenitor stem cells in veterinary and human ophthalmological applications. Stem Cell Reviews.

[13] Wang H, Zhang H, Huang B, Miao G, Yan X, Gao G, Yang L (2018). Mesenchymal stem cells reverse high-fat diet-induced non-alcoholic fatty liver disease through suppression of CD4+ T lymphocytes in mice. Molecular Medicine Reports.

[14] Tsuchiya A, Kojima Y, Ikarashi S, Seino S, Watanabe Y, Kawata Y, Terai S (2017). Clinical trials using mesenchymal stem cells in liver diseases and inflammatory bowel diseases. Inflammation and Regeneration.

[15] Marycz K, Michalak I, Kornicka K (2018). Advanced nutritional and stem cells approaches to prevent equine metabolic syndrome. Research in Veterinary Science.

[16] da Justa Pinheiro CH, de Queiroz JCF, Guimarães-Ferreira L, Vitzel KF, Nachbar RT, de Sousa LGO, Curi R (2012). Local injections of adipose-derived mesenchymal stem cells modulate inflammation and increase angiogenesis ameliorating the dystrophic phenotype in dystrophin-deficient skeletal muscle. Stem Cell Reviews and Reports.

[17] Fiore EJ, Mazzolini G, Aquino JB (2015). Mesenchymal stem/stromal cells in liver fibrosis: Recent findings, old/new caveats and future perspectives. Stem Cell Reviews and Reports.

[18] Marycz K, Kornicka K, Marędziak M, Golonka P, Nicpoń J (2016). Equine metabolic syndrome impairs adipose stem cells osteogenic differentiation by predominance of autophagy over selective mitophagy. Journal of Cellular and Molecular Medicine.

[19] Marycz K, Kornicka K, Basinska K, Czyrek A (2016). Equine metabolic syndrome affects viability, senescence, and stress factors of equine adipose-derived mesenchymal stromal stem cells: New insight into EqASCs Isolated from EMS horses in the context of their aging. Oxidative Medicine and Cellular Longevity.

[20] Kornicka Katarzyna, Houston Jenny, Marycz Krzysztof (2018). Dysfunction of Mesenchymal Stem Cells Isolated from Metabolic Syndrome and Type 2 Diabetic Patients as Result of Oxidative Stress and Autophagy may Limit Their Potential Therapeutic Use. Stem Cell Reviews and Reports.

[21] Pezzanite LM, Fortier LA, Antczak DF, Cassano JM, Brosnahan MM, Miller D, Schnabel LV (2015). Equine allogeneic bone marrow-derived mesenchymal stromal cells elicit antibody responses in vivo. Stem Cell Research & Therapy.

[22] Owens SD, Kol A, Walker NJ, Borjesson DL (2016). Allogeneic mesenchymal stem cell treatment induces specific alloantibodies in horses. Stem Cells International. Research article..

[23] Kornicka K, Marycz K, Marędziak M, Tomaszewski KA, Nicpoń J (2017). The effects of the DNA methyltranfserases inhibitor 5-Azacitidine on ageing, oxidative stress and DNA methylation of adipose derived stem cells. Journal of Cellular and Molecular Medicine.

[24] Yan X, Ehnert S, Culmes M, Bachmann A, Seeliger C, Schyschka L, Nussler AK (2014). 5-azacytidine improves the osteogenic differentiation potential of aged human adipose-derived mesenchymal stem cells by DNA demethylation. PLoS One.

[25] Švajger U, Jeras M (2012). Anti-inflammatory effects of resveratrol and its potential use in therapy of immune-mediated diseases. International Reviews of Immunology.

[26] Gülçin İ (2010). Antioxidant properties of resveratrol: A structure–activity insight. Innovative Food Science & Emerging Technologies.

[27] Aguirre L, Fernández-Quintela A, Arias N, Portillo MP (2014). Resveratrol: Anti-obesity mechanisms of action. Molecules (Basel, Switzerland).

[28] Bhullar KS, Hubbard BP (2015). Lifespan and healthspan extension by resveratrol. Biochimica et Biophysica Acta (BBA) - Molecular Basis of Disease.

[29] Kornicka K, Szłapka-Kosarzewska J, Śmieszek A, Marycz K (2019). 5-Azacytydine and resveratrol reverse senescence and ageing of adipose stem cells via modulation of mitochondrial dynamics and autophagy. Journal of Cellular and Molecular Medicine.

[30] Kornicka Katarzyna, Śmieszek Agnieszka, Węgrzyn Agnieszka, Röcken Michael, Marycz Krzysztof (2018). Immunomodulatory Properties of Adipose-Derived Stem Cells Treated with 5-Azacytydine and Resveratrol on Peripheral Blood Mononuclear Cells and Macrophages in Metabolic Syndrome Animals. Journal of Clinical Medicine.

[31] Kosolofski HR, Gow SP, Robinson KA (2017). Prevalence of obesity in the equine population of Saskatoon and surrounding area. The Canadian Veterinary Journal.

[32] Carter RA, Geor RJ, Burton Staniar W, Cubitt TA, Harris PA (2009). Apparent adiposity assessed by standardised scoring systems and morphometric measurements in horses and ponies. Veterinary Journal.

[33] Frank N, Walsh DM (2017). Repeatability of Oral sugar test results, glucagon-like Peptide-1 measurements, and serum high-molecular-weight adiponectin concentrations in horses. Journal of Veterinary Internal Medicine.

[34] Marędziak M, Marycz K, Lewandowski D, Siudzińska A, Śmieszek A (2015). Static magnetic field enhances synthesis and secretion of membrane-derived microvesicles (MVs) rich in VEGF and BMP-2 in equine adipose-derived stromal cells (EqASCs)-a new approach in veterinary regenerative medicine. In Vitro Cellular & Developmental Biology Animal.

[35] Marycz K, Kornicka K, Irwin-Houston JM, Weiss C (2018). Combination of resveratrol and 5-azacytydine improves osteogenesis of metabolic syndrome mesenchymal stem cells. Journal of Cellular and Molecular Medicine.

[36] Si Y, Zhao Y, Hao H, Liu J, Guo Y, Mu Y, Han W (2012). Infusion of mesenchymal stem cells ameliorates hyperglycemia in type 2 diabetic rats: Identification of a novel role in improving insulin sensitivity. Diabetes.

[37] Shree N, Bhonde RR (2017). Conditioned media from adipose tissue derived mesenchymal stem cells reverse insulin resistance in cellular models. Journal of Cellular Biochemistry.

[38] Xie Z, Hao H, Tong C, Cheng Y, Liu J, Pang Y, Han W (2016). Human umbilical cord-derived mesenchymal stem cells elicit macrophages into an anti-inflammatory phenotype to alleviate insulin resistance in type 2 diabetic rats. Stem Cells (Dayton, Ohio).

[39] Melief SM, Schrama E, Brugman MH, Tiemessen MM, Hoogduijn MJ, Fibbe WE, Roelofs H (2013). Multipotent stromal cells induce human regulatory T cells through a novel pathway involving skewing of monocytes toward anti-inflammatory macrophages. Stem Cells (Dayton, Ohio).

[40] Madrigal M, Rao KS, Riordan NH (2014). A review of therapeutic effects of mesenchymal stem cell secretions and induction of secretory modification by different culture methods. Journal of Translational Medicine.

[41] Zheng S, Yang J, Yang J, Tang Y, Shao Q, Guo L, Liu Q (2015). Transplantation of umbilical cord mesenchymal stem cells via different routes in rats with acute liver failure. International Journal of Clinical and Experimental Pathology.

[42] Huang B, Cheng X, Wang H, Huang W, la Ga Hu Z, Wang D, Zhang R (2016). Mesenchymal stem cells and their secreted molecules predominantly ameliorate fulminant hepatic failure and chronic liver fibrosis in mice respectively. Journal of Translational Medicine.

[43] Lee RH, Pulin AA, Seo MJ, Kota DJ, Ylostalo J, Larson BL, Prockop DJ (2009). Intravenous hMSCs improve myocardial infarction in mice because cells embolized in lung are activated to secrete the anti-inflammatory protein TSG-6. Cell Stem Cell.

[44] Eggenhofer E, Benseler V, Kroemer A, Popp FC, Geissler EK, Schlitt HJ, Hoogduijn MJ (2012). Mesenchymal stem cells are short-lived and do not migrate beyond the lungs after intravenous infusion. Frontiers in Immunology.

[45] Kim G, Eom YW, Baik SK, Shin Y, Lim YL, Kim MY, Chang SJ (2015). Therapeutic effects of mesenchymal stem cells for patients with chronic liver diseases: Systematic review and meta-analysis. Journal of Korean Medical Science.

[46] Wang L, Li J, Liu H, Li Y, Fu J, Sun Y, Wang F-S (2013). Pilot study of umbilical cord-derived mesenchymal stem cell transfusion in patients with primary biliary cirrhosis. Journal of Gastroenterology and Hepatology.

[47] Nam A., Han S.-M., Go D.-M., Kim D.-Y., Seo K.-W., Youn H.-Y. (2017). Long-Term Management with Adipose Tissue-Derived Mesenchymal Stem Cells and Conventional Treatment in a Dog with Hepatocutaneous Syndrome. Journal of Veterinary Internal Medicine.

[48] Liu Z, Meng F, Li C, Zhou X, Zeng X, He Y, Li T (2014). Human umbilical cord mesenchymal stromal cells rescue mice from acetaminophen-induced acute liver failure. Cytotherapy.

[49] Lindroos B, Suuronen R, Miettinen S (2011). The potential of adipose stem cells in regenerative medicine. Stem Cell Reviews.

[50] Bourebaba L, Röcken M, Marycz K (2019). Osteochondritis dissecans (OCD) in horses – Molecular background of its pathogenesis and perspectives for progenitor stem cell therapy. Stem Cell Reviews and Reports..

[51] Piryaei A, Valojerdi MR, Shahsavani M, Baharvand H (2011). Differentiation of bone marrow-derived mesenchymal stem cells into hepatocyte-like cells on nanofibers and their transplantation into a carbon tetrachloride-induced liver fibrosis model. Stem Cell Reviews and Reports.

